# Survival benefit of surgery with postoperative radiotherapy in locally advanced cervical adenocarcinoma: a population-based analysis

**DOI:** 10.1186/s12893-023-02203-3

**Published:** 2023-10-03

**Authors:** Xia Wang, Xiaojuan Lu, Junxing Chen, Hanjie Yi, Qiongyu Lan

**Affiliations:** 1 Department of Oncology, The Second Affiliated Hospital of Nanchang University, Nanchang University, No.1 Minde Street, Nanchang, 330000 Jiangxi Province People’s Republic of China; 2 Jiangxi Key Laboratory of Clinical Translational Cancer Research, Nanchang, Jiangxi Province China; 3 Radiation-Induced Heart Damage Institute of Nanchang University, Nanchang, Jiangxi Province China; 4 Department of Obstetrics and Gynecology, De’an County People’s Hospital, Jiujiang, Jiangxi Province China

**Keywords:** Cervical cancer, Adenocarcinoma, Surgery, Radiotherapy, Stages, Survival

## Abstract

**Background:**

The incidence of cervical adenocarcinoma (AC) has experienced a considerable increase in recent decades. Despite this, our understanding of the optimal management of locally advanced cervical AC remains limited. The present study sought to compare the clinical outcomes of radical hysterectomy with postoperative radiotherapy (PORT) and primary radiotherapy (RT) in patients with locally advanced cervical AC using the Surveillance, Epidemiology, and End Results (SEER) database.

**Methods:**

The data were extracted from the SEER database utilizing the SEER ∗ STAT software (version 8.4.0.1). The study included patients diagnosed with locally advanced cervical AC between 2004 and 2017 with adequate information available for analysis. Patients were assigned to either the Surgery + PORT or Primary RT group based on treatment modality, and their clinical characteristics were compared. Propensity score matching (PSM) was utilized to adjust for differences in baseline characteristics between groups. The primary endpoints of the study were overall survival (OS) and cancer-specific survival (CSS).

**Results:**

Of the 1363 patients who met the inclusion criteria, 302 (22.16%) underwent Surgery + PORT, while 1061 patients received Primary RT. The two groups differed significantly in terms of age, year of diagnosis, tumor size, grade, stage, T/N stage, and chemotherapy. PSM was performed to balance the baseline characteristics between the two groups, resulting in 594 patients being analyzed. After PSM, the Surgery + PORT group exhibited significantly improved survival rates. The 5-year OS rates were 69.7% (95% CI: 63.3%-76.9%) for the Surgery + PORT group and 60.9% (95% CI: 56.0%-66.3%) for the group receiving Primary RT (*p* = 0.002). The 5-year CSS rates for the two groups were 70.7% (95% CI: 64.3%-77.8%) and 66.2% (95% CI: 61.3%-71.5%), respectively (*p* = 0.049). Multivariate analysis revealed that Surgery + PORT was an independent favorable prognostic factor for OS (HR = 0.60, *p* = 0.001) and CSS (HR = 0.69, *p* = 0.022). Although the combined approach of surgery and PORT resulted in a favorable impact on OS in patients aged 65 years or older (HR = 0.57, *p* = 0.048), it did not result in a statistically significant improvement in CSS in the same age group (HR = 0.56, *p* = 0.087). Similarly, the combined treatment did not yield a statistically significant increase in either OS (HR = 0.78, *p* = 0.344) or CSS (HR = 0.89, *p* = 0.668) in patients with tumors larger than 60 mm.

**Conclusion:**

The present study demonstrated that Surgery + PORT was associated with improved OS and CSS in patients with locally advanced cervical AC when compared to Primary RT. As such, Surgery + PORT may be a preferable therapeutic option for carefully selected patients with cervical AC. These findings offer valuable insight into the management of locally advanced cervical AC and may assist in personalized treatment decisions.

**Supplementary Information:**

The online version contains supplementary material available at 10.1186/s12893-023-02203-3.

## Introduction

Cervical cancer ranks fourth among the most frequently occurring types of cancer in women, accounting for an estimated 604,127 cases and 341,831 deaths worldwide in 2020 [1]. This disease is a critical global health issue, with squamous cell carcinoma (SCC) representing the most common histologic subtype. However, the incidence of adenocarcinoma (AC) has been steadily rising in recent decades, particularly among younger women, and now comprises approximately 20–25% of all cervical cancers [2, 3]. This trend is concerning because AC is associated with worse outcomes and is less responsive to standard treatments compared to SCC [3, 4]. Despite this, there are currently no clear differences in treatment between AC and SCC outlined in the National Comprehensive Cancer Network guidelines [5].

Early-stage cervical cancer is primarily treated with surgery or radiotherapy (RT). Surgery is generally reserved for early-stage disease, fertility preservation, and smaller lesions such as stage IA, IB1, IB2, and selected IIA1 [5, 6]. Several studies have demonstrated that surgery is the optimal local treatment modality for patients with early-stage cervical AC [3, 7]. Traditionally, locally advanced disease has included patients with stage IIB to IVA disease. However, an increasing number of oncologists now classify patients with IB3 and IIA2 disease as advanced disease. The panel agrees that concurrent chemoradiation (CCRT) is usually the primary treatment of choice for stages IB3 to IVA disease based on the results of 5 randomized clinical trials [5, 8, 9]. Although few studies have evaluated treatment specifically for AC, they are typically treated in a similar manner to SCC [4, 10, 11]. Hence, there is currently a dearth of level 1 evidence to provide guidance for managing patients with locally advanced cervical AC, resulting in limited understanding of the optimal approach to such management. The ideal management strategy for cervical AC continues to be a matter of debate among healthcare professionals, particularly with regard to whether stages IB3 to IVA of cervical AC should be managed differently than SCC, and which therapeutic options should be considered.

Therefore, we conducted a retrospective study using propensity-matching to evaluate the impact of two treatment options, radical hysterectomy followed by PORT and Primary RT, on locally advanced cervical AC. The study relied on data obtained from the Surveillance, Epidemiology, and End Results (SEER) database, a population-based registry that covers approximately 34.6% of the United States (US) population and provides extensive epidemiological information on cancer cases [12]. The primary endpoints of this study were overall survival (OS) and cancer-specific survival (CSS). We anticipate that the findings of this study will shed light on the optimal therapeutic strategy for patients with locally advanced cervical AC and identify the target population most likely to benefit from Surgery + PORT treatment.

## Patients and methods

### Data sources

The data were obtained from the SEER database utilizing the SEER ∗ STAT software (version 8.4.0.1). The SEER program, administered by the National Cancer Institute in the United States, aggregates data from 18 population-based cancer registries, encompassing about 34.6% of the US population, and thus, offers a representative sample of cancer cases nationwide. The SEER data are accessible to the public and can be obtained through the SEER website.

### Study population and definition

**Fig. 1:**
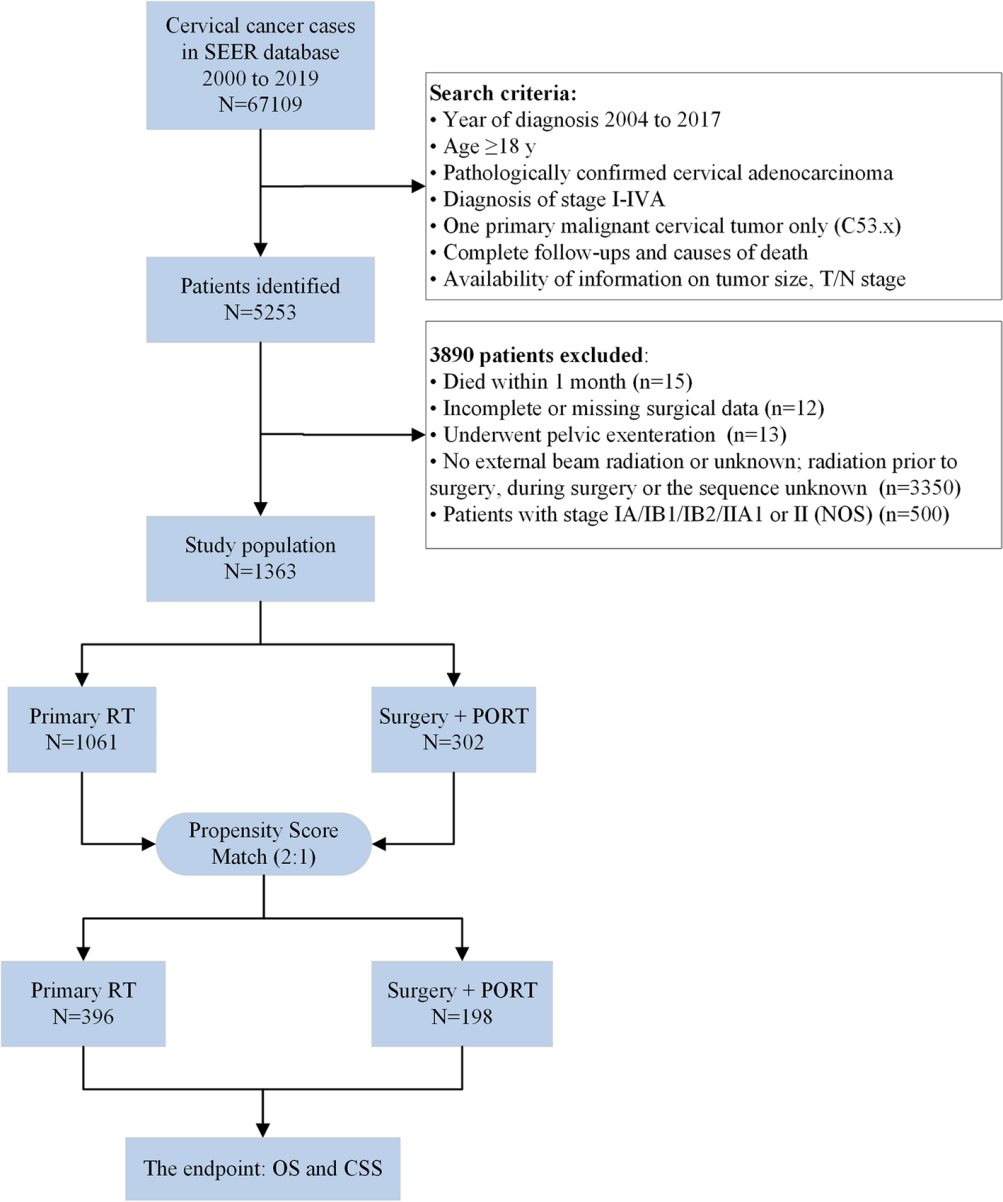
Flow chart of the screened patients. SEER Surveillance, Epidemiology, and End Results, RT radiotherapy, PORT postoperative radiotherapy, OS overall survival, CSS cancer-specific survival

We herein present an investigation into patients diagnosed with cervical cancer during the period between 2004 and 2017. The study's primary inclusion criteria encompassed a range of prerequisites, including the following: (1) age exceeding 18 years; (2) confirmation of pathologically identified cervical AC, classified under the ICD-O-3 codes of 8140/3, 8144/3, 8147/3, 8200/3, 8210/3, 8241/3, 8244/3, 8255/3, 8260/3, 8261/3, 8262/3, 8263/3, 8310/3, 8313/3, 8323/3, 8380/3, 8382/3, 8384/3, 8430/3, 8441/3, 8460/3, 8461/3, 8480/3, 8481/3, 8482/3, and 8490/3 [13, 14]; (3) diagnosis of locally advanced cervical cancer, specifically stage IB3/IIA2-IVA; (4) one primary malignant cervical cancer only (C53.0–53.1 and C53.8–53.9); (5) complete follow-ups and causes of death; (6) available information regarding tumor size and stage; and (7) information regarding treatment modalities employed, including surgical interventions and RT. Key exclusion criteria comprised patients with incomplete registration data, those who perished within one month, and those who received no local treatment or underwent solely surgical interventions. The cohort was subsequently bifurcated into two distinct groups based on the modality of treatment, specifically RT alone (Primary RT group) and radical surgery (modified radical or extended hysterectomy) followed by RT (Surgery + PORT group). For more detailed information regarding the patient selection process, please refer to Fig. 1.

The present study gathered data from the SEER database utilizing the SEER*Stat software. The variables extracted from the SEER database included age at diagnosis, race and ethnicity, stage at diagnosis (conversion of staging according to 2018 International Federation of Gynecology and Obstetrics [FIGO] staging system), histologic subtype, year of diagnosis, tumor size, pathologic grade, surgical procedure, radiation recode, chemotherapy, survival months, vital status recode, cause of death, and cause-specific death classification.

The study's primary endpoints were OS and CSS. The definition of OS refers to the duration from the date of cancer diagnosis to either the date of death from any cause or the conclusion of the follow-up period, which concluded on December 31, 2019. CSS was described as the period between the diagnosis of cervical cancer and death due to that particular malignancy.

### Statistical analysis

The data were reported as median (range) or n (%). Comparisons of clinicopathological characteristics between groups were conducted using Fisher’s exact test for categorical variables and the two-sample t-test or Mann–Whitney U-test for continuous variables, as appropriate. The X-tile program (version 3.6.1) was utilized to determine the cut-off values for continuous variables, namely age and tumor size. Propensity score matching (PSM) with a matching tolerance of 0.03 was employed to balance baseline characteristics between the Primary RT and Surgery + PORT groups. OS was assessed using Kaplan–Meier plots and the Cox log-rank test. Multivariate survival analyses were conducted using the Cox proportional hazards regression model. To evaluate risk factors for CSS with non-cancer deaths as the competing risk, univariate and multivariate analyses were performed using the competing risk regression (Fine and Gray method) [15]. Variables with *p* values ≤ 0.20 in the univariate analysis were included in the multivariate analysis. Statistical significance was set at *p* values < 0.05. The data were subjected to analysis using R software packages (http://www.R-project.org, The R Foundation) as well as Empower Stats software (http://www.empowerstats.com, X&Y Solutions, Inc., Boston, MA).

## Results

### Patient characteristics

In accordance with our inclusion criteria, a total of 1363 patients were deemed eligible and consequently incorporated into our study cohort. A detailed schematic of the selection process can be found in Fig. 1. Of these patients, 302 (22.16%) underwent radical hysterectomy followed by PORT, while 1061 patients received RT alone (Table 1). Simultaneously administered chemotherapy was received by a majority of patients, with 250 (82.78%) and 935 (88.12%) individuals receiving chemotherapy in the two respective groups. Notably, the distribution of patient demographics, including age, year of diagnosis, tumor size, grade, FIGO stage, T/N stage and chemotherapy, varied significantly between the two aforementioned groups. There was no discernible difference in the racial distribution between the two groups. By employing PSM at a 2:1 ratio, a study cohort consisting of 594 patients was identified. Following PSM, there were no statistically significant disparities in clinicopathological patient features between the Primary RT group and Surgery + PORT group, as evinced in Table 1.

**Table 1:** The baseline clinical characteristics of enrolled patients with stage IB3/IIA2-IVA cervical adenocarcinoma before and after PSM

Clinical parameters	Before PSM			After PSM		
	**Primary RT** **(*N* = 1061)**	**Surgery + PORT** **(*N* = 302)**	***p*-value**	**Primary RT** **(*N* = 396)**	**Surgery + PORT** **(*N* = 198)**	***p*-value**
**Age, years (range)**	50 (21–96)	46 (22–88)	< 0.001*	50 (24–95)	48 (22–88)	0.104
**Race**			0.162			0.600
Black	87 (8.20%)	15 (4.97%)		28 (7.07%)	11 (5.56%)	
White	835 (78.70%)	244 (80.79%)		304 (76.77%)	159 (80.30%)	
Others or Unknown	139 (13.10%)	43 (14.24%)		64 (16.16%)	28 (14.14%)	
**Year of diagnosis**			0.027*			0.476
2004–2008	282 (26.58%)	97 (32.12%)		94 (23.74%)	56 (28.28%)	
2009–2013	362 (34.12%)	111 (36.75%)		150 (37.88%)	72 (36.36%)	
2014–2017	417 (39.30%)	94 (31.13%)		152 (38.38%)	70 (35.35%)	
**Tumor size, mm**			< 0.001*			0.134
< = 40	274 (25.82%)	153 (50.66%)		128 (32.32%)	66 (33.33%)	
> 40, < = 60	480 (45.24%)	114 (37.75%)		179 (45.20%)	101 (51.01%)	
> 60	307 (28.93%)	35 (11.59%)		89 (22.47%)	31 (15.66%)	
**Grade**			< 0.001*			0.122
Well differentiated	137 (12.91%)	52 (17.22%)		71 (17.93%)	35 (17.68%)	
Moderately differentiated	339 (31.95%)	139 (46.03%)		162 (40.91%)	82 (41.41%)	
Poorly differentiated	251 (23.66%)	80 (26.49%)		80 (20.20%)	54 (27.27%)	
Undifferentiated; anaplastic	47 (4.43%)	12 (3.97%)		27 (6.82%)	11 (5.56%)	
Unknown	287 (27.05%)	19 (6.29%)		56 (14.14%)	16 (8.08%)	
**FIGO stage**			< 0.001*			0.925
IB3/IIA2-IIB	573 (54.01%)	104 (34.44%)		192 (48.48%)	99 (50.00%)	
III	464 (43.73%)	195 (64.57%)		198 (50.00%)	96 (48.48%)	
IIIA	27 (2.54%)	2 (0.66%)		5 (1.26%)	2 (1.01%)	
IIIB	75 (7.07%)	1 (0.33%)		23 (5.81%)	1 (0.51%)	
IIIC	351 (33.08%)	189 (62.58%)		168 (42.42%)	90 (45.45%)	
III, NOS	11 (1.04%)	3 (0.99%)		2 (0.51%)	3 (1.52%)	
IVA	24 (2.26%)	3 (0.99%)		6 (1.52%)	3 (1.52%)	
**T stage**			< 0.001*			0.235
T1	338 (31.86%)	167 (55.30%)		155 (39.14%)	77 (38.89%)	
T2	512 (48.26%)	115 (38.08%)		179 (45.20%)	101 (51.01%)	
T3	187 (17.62%)	17 (5.63%)		56 (14.14%)	17 (8.59%)	
T4	24 (2.26%)	3 (0.99%)		6 (1.52%)	3 (1.52%)	
**N stage**			< 0.001*			0.482
N0	696 (65.60%)	113 (37.42%)		228 (57.58%)	108 (54.55%)	
N1	365 (34.40%)	189 (62.58%)		168 (42.42%)	90 (45.45%)	
**Chemotherapy**			0.015*			0.593
No	126 (11.88%)	52 (17.22%)		67 (16.92%)	37 (18.69%)	
Yes	935 (88.12%)	250 (82.78%)		329 (83.08%)	161 (81.31%)	

*PSM* propensity score-matching, *RT* radiotherapy, *PORT* postoperative radiotherapy, *FIGO* the International Federation of Gynecology and Obstetrics, *NOS* Not Otherwise Specified

^*^ *p* < 0.05 was considered significant. Data represent as median (range) or n (%)

### Survival outcomes

**Fig. 2:**
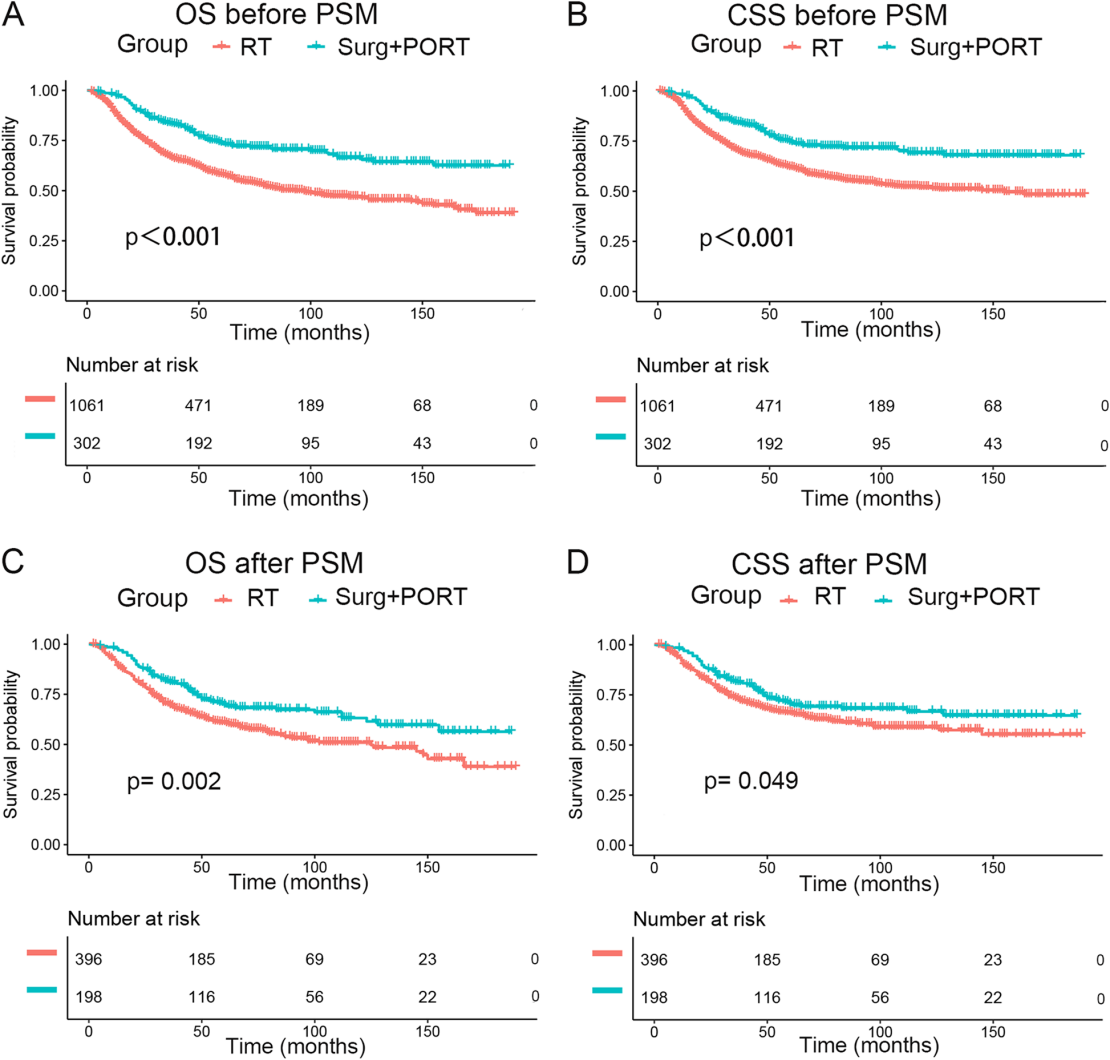
Survival curves according local treatment before and after PSM: **A** OS curves before PSM; **B** CSS curves before PSM; **C** OS curves after PSM; **D** CSS curves after PSM. OS overall survival, CSS cancer-specific survival, PSM propensity score matching, RT radiotherapy, Sury Surgery, PORT postoperative radiotherapy

Prior to PSM, the 5-year OS rates for patients receiving Primary RT versus Surgery + PORT were 58.3% (95% CI: 55.1%-61.5%) and 73.6% (95% CI: 68.6%-79.1%), respectively (*p* < 0.001) (Fig. 2A). The 5-year CSS rates for Primary RT and Surgery + PORT were 61.9% (95% CI: 58.8%-65.2%) and 74.3% (95% CI: 69.2%-79.7%), respectively (*p* < 0.001) (Fig. 2B).

After PSM, the Surgery + PORT group continued to demonstrate significantly improved survival rates. Specifically, the 5-year OS rates for the two groups were 60.9% (95% CI: 56.0%-66.3%) and 69.7% (95% CI: 63.3%-76.9%), respectively (*p* = 0.002) (Fig. 2C). The 5-year CSS rates were 66.2% (95% CI: 61.3%-71.5%) and 70.7% (95% CI: 64.3%-77.8%), respectively (*p* = 0.049) (Fig. 2D).

### Prognostic factors

Following PSM, the univariate analysis revealed that age, race, grade, FIGO stage, T stage, N stage, and local treatment were significantly associated with OS (Table 2). Moreover, the variables linked with CSS included age, race, year of diagnosis, tumor size, grade, FIGO stage, T stage, and N stage (Table 3).

**Table 2:** Univariate and multivariate analyses of factors for OS after PSM

Variable name	Univariate analysis (*N* = 594)				Multivariate analysis (*N* = 594)			
	**HR**	**95% CI for HR**		***p* value**	**HR**	**95% CI for HR**		***p* value**
**Age, years**								
< 65	Reference				Reference			
> = 65	2.36	1.74	3.20	< 0.001	2.21	1.58	3.10	< 0.001
**Race**								
Black	Reference				Reference			
White	0.54	0.35	0.83	0.006	0.51	0.33	0.81	0.004
Others or Unknown	0.73	0.44	1.22	0.233	0.83	0.49	1.40	0.478
**Year of diagnosis**								
2004–2008	Reference				Reference			
2009–2013	0.87	0.64	1.19	0.377	0.80	0.58	1.10	0.168
2014–2017	0.73	0.51	1.05	0.087	0.69	0.48	0.99	0.047
**Tumor size, mm**								
< = 40	Reference				Reference			
> 40, < = 60	1.15	0.85	1.55	0.370	1.66	1.22	2.28	0.001
> 60	1.32	0.92	1.88	0.130	1.47	1.01	2.15	0.045
**Grade**								
Well differentiated	Reference				Reference			
Moderately differentiated	1.26	0.82	1.94	0.293	1.21	0.78	1.88	0.392
Poorly differentiated	1.93	1.23	3.02	0.004	1.66	1.04	2.65	0.033
Undifferentiated; anaplastic	2.81	1.62	4.88	< 0.001	2.28	1.27	4.10	0.006
Unknown	2.72	1.68	4.43	< 0.001	1.81	1.09	3.00	0.023
**FIGO stage**								
IB3/IIA2-IIB	Reference				Reference			
III	2.17	1.65	2.85	< 0.001	0.80	0.40	1.60	0.531
IVA	3.80	1.75	8.24	0.001	5.06	2.20	11.64	< 0.001
**T stage**								
T1	Reference				Reference			
T2	1.59	1.17	2.15	0.003	1.60	1.17	2.21	0.004
T3	2.91	1.98	4.28	< 0.001	3.31	2.01	5.45	< 0.001
T4	3.68	1.68	8.04	0.001	**—**	**—**	**—**	**—**
**N stage**								
N0	Reference				Reference			
N1	1.88	1.45	2.44	< 0.001	2.38	1.27	4.44	0.007
**Chemotherapy**								
No	Reference				**—**			
Yes	1.13	0.80	1.59	0.498	**—**	**—**	**—**	**—**
**Local treatment**								
Primary RT	Reference				Reference			
Surgery + PORT	0.64	0.48	0.86	0.003	0.60	0.44	0.81	0.001

*p* < 0.05 was considered significant

Model adjusted for multivariate analysis: Age, Race, Year of diagnosis, Tumor size, Grade, FIGO stage, T stage, N stage, Local treatment

*OS* overall survival, *PSM* propensity score-matching, *HR* hazard ratio, *CI* confidence interval, *FIGO* the International Federation of Gynecology and Obstetrics, *RT* radiotherapy, *PORT* postoperative radiotherapy

**Table 3:** Univariate and multivariate analyses of factors for CSS after PSM using a Fine-Gray hazard model

Variable name	Univariate analysis (*N* = 594)				Multivariate analysis (*N* = 594)			
	**HR**	**95% CI for HR**		***p* value**	**HR**	**95% CI for HR**		***p* value**
**Age, years**								
< 65	Reference				Reference			
> = 65	1.76	1.22	2.54	0.002	1.77	1.20	2.62	0.004
**Race**								
Black	Reference				Reference			
White	0.53	0.32	0.86	0.010	0.49	0.29	0.80	0.005
Others or Unknown	0.79	0.45	1.40	0.422	0.88	0.50	1.55	0.655
**Year of diagnosis**								
2004–2008	Reference				Reference			
2009–2013	0.78	0.56	1.10	0.154	0.74	0.52	1.05	0.087
2014–2017	0.49	0.33	0.71	< 0.001	0.61	0.41	0.91	0.014
**Tumor size, mm**								
< = 40	Reference				Reference			
> 40, < = 60	1.26	0.89	1.78	0.187	1.83	1.29	2.59	0.001
> 60	1.63	1.09	2.44	0.018	1.68	1.11	2.53	0.014
**Grade**								
Well differentiated	Reference				Reference			
Moderately differentiated	1.29	0.80	2.07	0.291	1.13	0.71	1.81	0.608
Poorly differentiated	2.02	1.24	3.31	0.005	1.47	0.89	2.43	0.133
Undifferentiated; anaplastic	2.57	1.41	4.71	0.002	2.18	1.16	4.11	0.016
Unknown	2.54	1.48	4.35	0.001	1.71	0.99	2.96	0.055
**FIGO stage**								
IB3/IIA2-IIB	Reference				Reference			
III	2.53	1.85	3.46	< 0.001	0.80	0.37	1.72	0.569
IVA	3.79	1.75	8.22	0.001	6.01	2.43	14.85	< 0.001
**T stage**								
T1	Reference				Reference			
T2	1.83	1.29	2.61	0.001	1.84	1.29	2.63	0.001
T3	3.38	2.17	5.26	< 0.001	3.87	2.27	6.60	< 0.001
T4	3.67	1.66	8.07	0.001	—	—	—	—
**N stage**								
N0	Reference				Reference			
N1	2.20	1.64	2.95	< 0.001	2.66	1.34	5.30	0.005
**Chemotherapy**								
No	Reference				—			
Yes	1.18	0.78	1.78	0.426	—	—	—	—
**Local treatment**								
Primary RT	Reference				Reference			
Surgery + PORT	0.73	0.54	1.00	0.050	0.69	0.50	0.95	0.022

Model adjusted for multivariate analysis: Age, Race, Year of diagnosis, Tumor size, Grade, FIGO stage, T stage, N stage, Local treatment

*CSS* cancer-specific survival, *PSM* propensity score-matching, *HR* hazard ratio, *CI* confidence interval, *FIGO* the International Federation of Gynecology and Obstetrics, *RT* radiotherapy, *PORT* postoperative radiotherapy

*p* < 0.05 was considered significant

Upon conducting multivariable analysis, surgery with PORT emerged as an independent prognostic factor for OS (HR = 0.60, *p* = 0.001) and CSS (HR = 0.69, *p* = 0.022), along with age, race, year of diagnosis, tumor size, grade, FIGO stage, T stage, and N stage, as shown in Table 2 and Table 3. Furthermore, the univariate and multivariate analyses utilizing unmatched data yielded results consistent with those obtained from matched data, demonstrating that surgery with PORT is an independent prognostic factor, as showcased in Table S1 and Table S2.

### Subgroup analysis before PSM

**Fig. 3:**
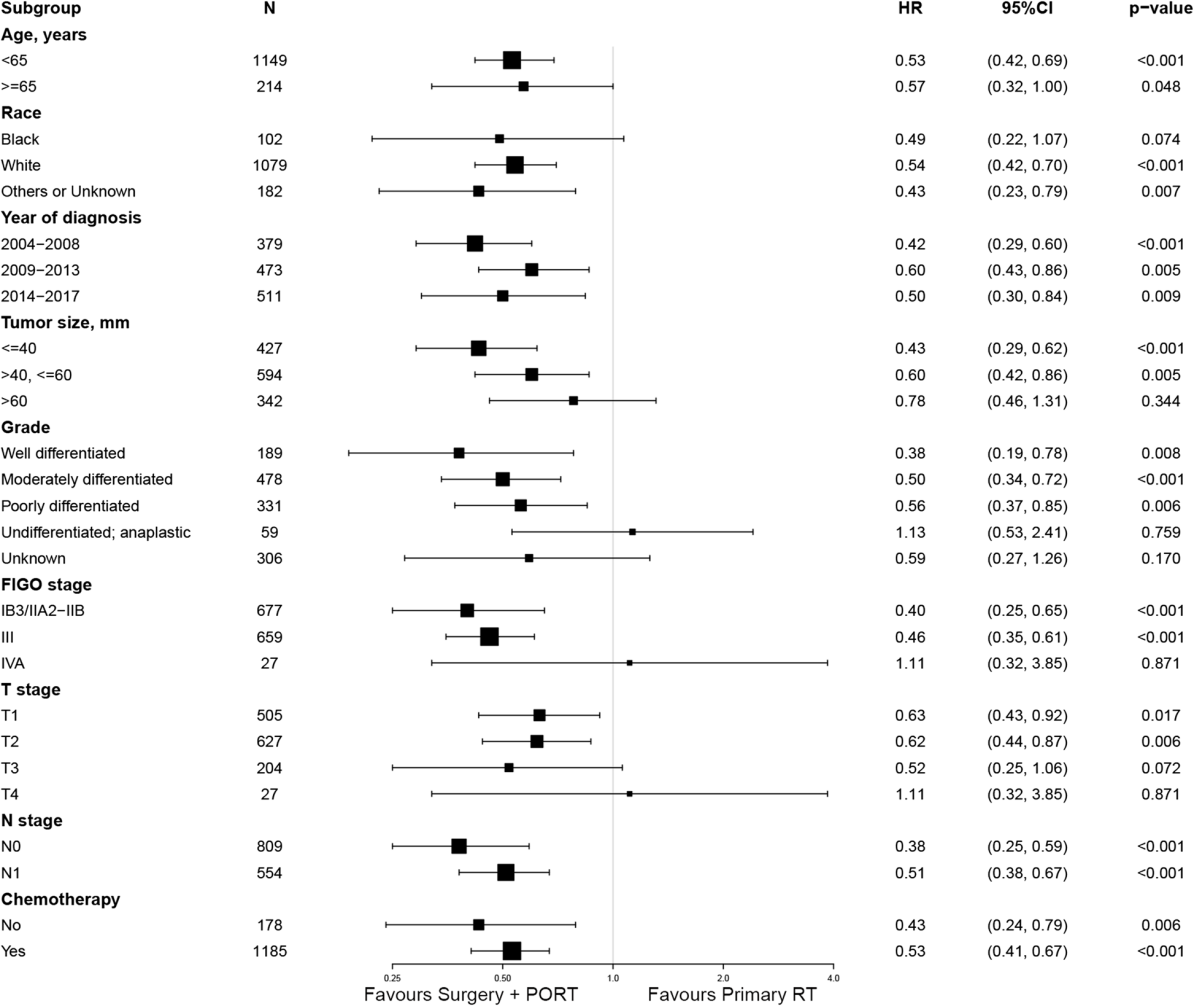
Forest plot of the subgroup analysis concerning overall survival. HR hazard ratio, CI confidence interval, FIGO the International Federation of Gynecology and Obstetrics, PORT postoperative radiotherapy, RT radiotherapy

**Fig. 4:**
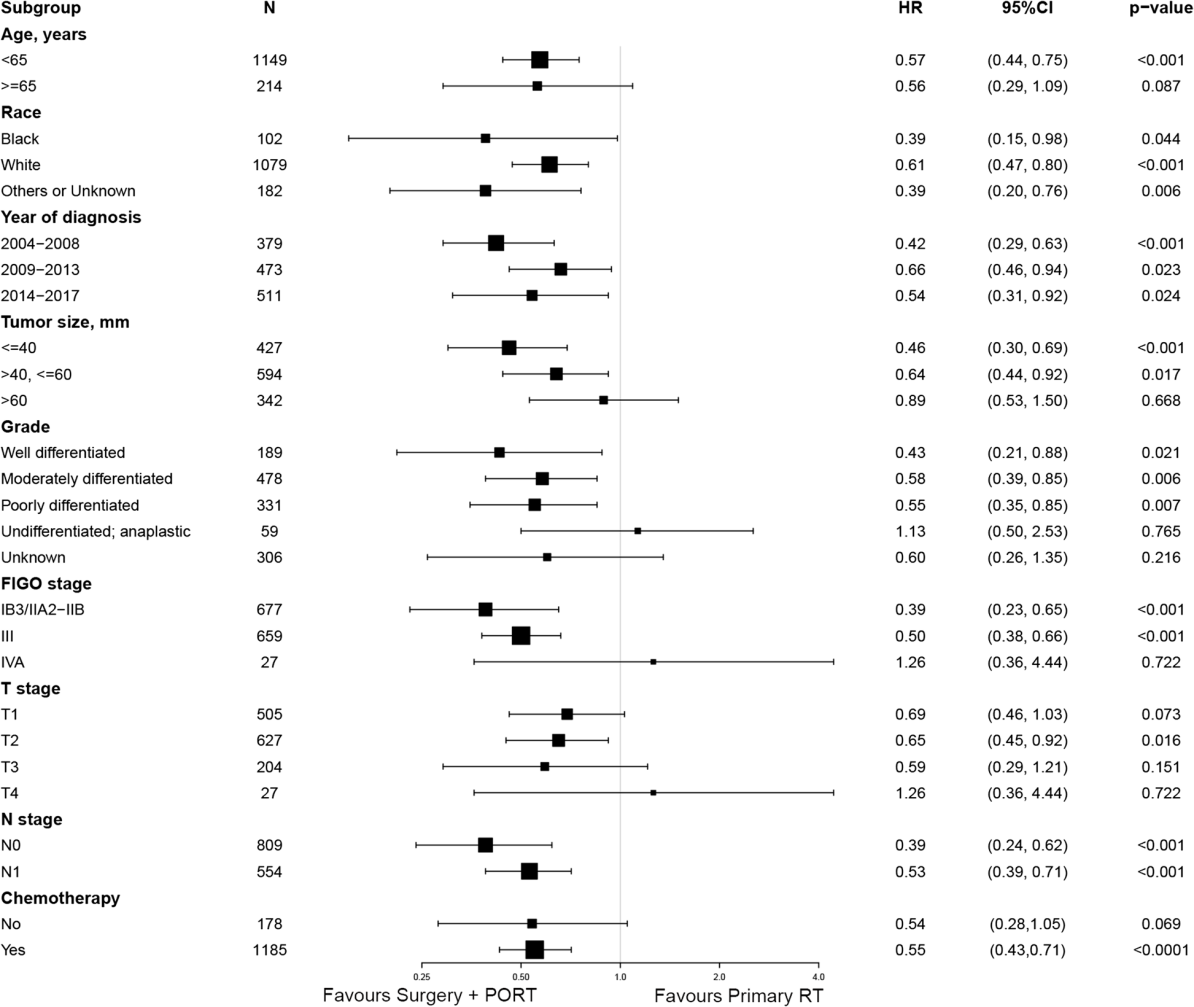
Forest plot of the subgroup analysis concerning cancer-specific survival. HR hazard ratio, CI confidence interval, FIGO the International Federation of Gynecology and Obstetrics, PORT postoperative radiotherapy, RT radiotherapy

Our subgroup analysis revealed that the discrepancy in OS and CSS between the two cohorts remained consistent across most cervical cancer subgroups (Figs. 3 and 4). Patients who received surgical intervention followed by PORT exhibited superior outcomes in terms of both OS and CSS, compared to those who underwent RT alone.

It is noteworthy that both age and tumor size are significant prognostic factors, demonstrating a close association with OS and CSS in patients, as revealed by the findings presented in Tables 2 and 3, respectively. Patients aged 65 years or older or those with a tumor size exceeding 40 mm display a comparatively unfavorable prognosis, as depicted in Fig. S1(A-D). Although the combined approach of surgery and PORT resulted in a favorable impact on OS in patients aged 65 years or older (HR = 0.57, *p* = 0.048), it did not engender a statistically significant improvement in CSS in the same age group (HR = 0.56, *p* = 0.087), as illustrated in Figs. 3 and 4. Similarly, the integration of radical hysterectomy with PORT did not yield a statistically significant increase in either OS (HR = 0.78, *p* = 0.344) or CSS (HR = 0.89, *p* = 0.668) in patients with tumors larger than 60 mm (Figs. 3 and 4).

## Discussion

Cervical AC is a subtype of cervical cancer that is less prevalent than SCC but has been experiencing a rise in incidence in recent years [2, 3]. The epidemiological, clinicopathological, and molecular features, as well as the treatment response and prognosis, of AC of the uterine cervix diverge from those of SCC, as demonstrated by numerous studies [4, 16]. Most studies suggest that AC is linked to a more unfavorable prognosis compared to SCC [7, 17–20]. In a comprehensive investigation utilizing data from the SEER database, which comprised an astounding 33,148 patients diagnosed with cervical cancer, it was revealed that individuals with AC had a higher risk of mortality (HR = 1.12, 95% CI:1.07–1.18, *p* < 0.001) than their SCC counterparts [20]. Upon stratification by stage, a disheartening prognosis was observed for patients with stage II-III AC affliction, as compared to those with SCC (HR = 1.24, 95% CI:1.14–1.36, *p* < 0.001), while no significant difference was noted among individuals with stage I or IV disease subgroups. The use of SCC treatments for cervical AC may be debatable, but optimal treatments for cervical AC at different clinical stages remain unknown due to a lack of sufficient evidence.

According to extant recommendations for the management of locally advanced cervical cancer, the preferred therapeutic strategy involves CCRT, with surgical intervention serving only as a secondary modality [5, 8, 9]. For women with locoregionally advanced cervical SCC, CCRT has been the treatment of choice at most institutions. A compelling counterargument to utilizing surgical intervention as the primary therapeutic modality for this patient population is the notable probability of requiring adjuvant therapy, resulting in elevated risks of adverse reactions and superfluous expenses [7, 21–23]. This holds particular relevance for the majority of patients with locally advanced diseases exhibiting high incidence of unfavourable histopathological parameters, where adjuvant CCRT following surgical intervention is commonly advocated [21–23]. However, it is unclear whether surgery should be considered as a treatment option for locally advanced cervical AC.

In this investigation, we utilized the SEER database to evaluate the impact of two treatment options, radical hysterectomy followed by PORT and RT alone, on survival outcomes in individuals with locally advanced cervical AC. Our analysis revealed that patients who underwent surgery followed by PORT exhibited significantly higher 5-year OS and CSS rates in both the pre-match and matched cohorts after adjustment in multivariate analysis when compared to those who received RT alone. The cohort that underwent Surgery + PORT demonstrated an OS rate of 69.7% and a CSS rate of 70.7%, while the Primary RT group exhibited an OS rate of 60.9% and a CSS rate of 66.2% in the matched cohorts. These results imply that surgery + PORT may represent a more efficacious therapeutic strategy for managing locally advanced cervical AC and carry significant implications for clinical practice. Our findings are consistent with a previous study which demonstrated that surgery represents the most efficacious local treatment modality for individuals presenting with advanced clinical stages [7]. However, the groupings utilized in this previous investigation lacked rigor [7]. Specifically, the first group comprised patients who underwent radical surgical intervention, with uncertain postoperative adjuvant therapy, while the second group consisted of patients who received CCRT, with some individuals subsequently undergoing surgical treatment.

The less encouraging outcomes observed for RT may be attributable to advanced-stage cervical AC exhibiting elevated radioresistance, large sizes, extensive metastases, and high depths of invasion, which cannot be overcome by even curative definitive CCRT with concurrent cisplatin-based chemotherapy [7, 24]. Nearly one in four (24%) of patients with locally advanced and higher stage cervical cancer may experience central persistence of disease following CCRT, and more aggressive surgery in patients exhibiting minimal central residual disease after (chemo)radiation does not improve survival and should not be recommended [25]. By performing surgery as the primary treatment modality, it is possible to remove substantial volumes of radioresistant AC tumors, which may result in superior OS and CSS when compared to definitive CCRT [7, 24].

The survival outcomes of patients with AC decrease significantly as the size of the tumor increases. Tumor size greater than 40 mm is a significant adverse prognostic factor for survival, as demonstrated by previous studies [3, 26, 27], which is consistent with our findings. In our study, patients with tumors larger than 40 mm or 60 mm exhibited a poorer prognosis than those with tumors 40 mm or smaller. It is noteworthy that subgroup analysis revealed no significant difference in OS and CSS outcomes between patients with tumor size greater than 60 mm who underwent surgery with PORT compared to those treated with Primary RT alone. Similarly, previous studies have established a correlation between older age and poor outcomes, a finding that is consistent with our results [3, 28]. In our study, surgery with PORT was not associated with improved CSS in patients older than 65 years. Therefore, for patients with tumor size exceeding 60 mm or who are 65 years of age or older, the selection of local treatment modalities should be based on careful consideration of individualized patient factors.

Our study possesses several notable strengths, with the foremost among them being the implementation of PSM to adjust for potential confounding factors and the utilization of a sizable sample obtained from the SEER database. Nonetheless, our investigation is not devoid of limitations. Notably, a restricted subset comprising 178 individuals (13.1%) did not undergo chemotherapy, which could have influenced the efficacy of RT. Furthermore, we encountered obstacles in assessing the adverse effects of treatment modalities for the Surgery + PORT and RT groups due to inadequate data availability in the SEER database. It is also crucial to underscore that, owing to inherent constraints within the SEER database, our capacity to integrate precise particulars pertaining to the modalities of radical hysterectomy undertaken, the determination of residual tumor presence or absence, the method and dimensions of lymph node metastasis imaging assessment, and the specific radiotherapy techniques employed was regrettably restricted. The inclusion of such data, had it been accessible, might have imparted invaluable depth to our analytical endeavors. Lastly, despite the relatively abundant dataset, the underrepresentation of undifferentiation and stage T3 patients precluded our ability to perform subgroup analyses.

In conclusion, our population-based analysis provides evidence that surgery followed by PORT is associated with better OS and CSS than Primary RT alone for the management of locally advanced cervical AC. This finding may represent a more efficacious therapeutic strategy for managing locally advanced cervical AC and has significant implications for clinical practice. However, careful consideration of individualized patient factors such as tumor size and patient age should be taken into account when selecting the optimal local treatment modalities. Further well-designed prospective studies are warranted to determine the long-term outcomes of surgery with PORT, and to assess its feasibility and safety.

### Supplementary Information

**Additional file 1: Figure S1.** Survival curves stratified by age and tumor size prior to PSM. The curves include OS curves stratified by age (**A**) and tumor size (**C**), as well as CSS curves stratified by age (**B**) and tumor size (**D**). OS overall survival, CSS cancer-specific survival, PSM propensity score matching.

**Additional file 2:Table S1.** Univariate and multivariate analyses of factors for OS before PSM.

**Additional file 3:** **Table S2.** Univariate and multivariate analyses of factors for CSS before PSM using a Fine-Gray hazard model.

## Data Availability

The data files used in this study were directly downloaded from the SEER website (https://seer.cancer.gov).

## Abbreviations

AC: Adenocarcinoma
PORT: Postoperative radiotherapy
RT: Radiotherapy
SEER: Surveillance, Epidemiology, and End Results
PSM: Propensity score matching
OS: Overall survival
CSS: Cancer-specific survival
SCC: Squamous cell carcinoma
CCRT: Concurrent chemoradiation
US: United States
FIGO: International Federation of Gynecology and Obstetrics
NOS: Not Otherwise Specified
