# Supplementary figures and images for: Survival benefit of surgery with postoperative radiotherapy in locally advanced cervical adenocarcinoma: a population-based analysis
Source: BMC Surg. 2023 Oct 3;23:299. doi: 10.1186/s12893-023-02203-3 (PMC10548725; doi:10.1186/s12893-023-02203-3)

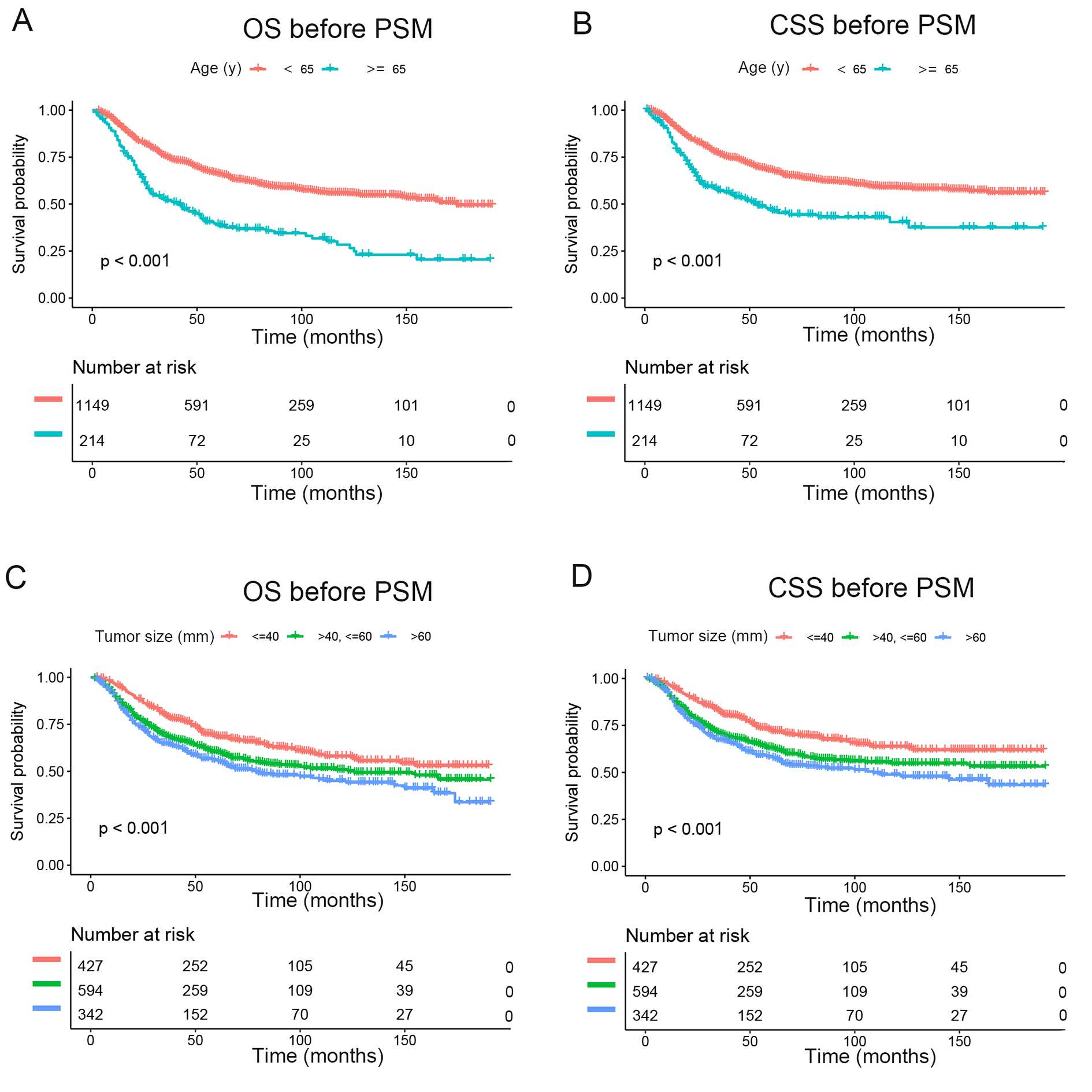

Supplement: Supplementary file 1 — Additional file 1: Figure S1. Survival curves stratified by age and tumor size prior to PSM. The curves include OS curves stratified by age (A) and tumor size (C), as well as CSS curves stratified by age (B) and tumor size (D). OS overall survival, CSS cancer-specific survival, PSM propensity score matching. [file 12893_2023_2203_MOESM1_ESM.tif]
